# Long-term experience of hyperbaric oxygen therapy for refractory radio- or chemotherapy-induced haemorrhagic cystitis

**DOI:** 10.1186/s12894-015-0035-4

**Published:** 2015-05-08

**Authors:** Stephan Degener, Alexander Pohle, Hartmut Strelow, Michael J Mathers, Jürgen Zumbé, Stephan Roth, Alexander S Brandt

**Affiliations:** Department of Urology, HELIOS Medical Center Wuppertal, University of Witten/Herdecke, Heusnerstrasse 40, 42283 Wuppertal, Germany; Department of Urology, Medical Center Leverkusen, Am Gesundheitspark 11, 51375 Leverkusen, Germany; Institute of Hyperbaric Oxygen (HBO), University Hospital Düsseldorf, Moorenstrasse 5, 40225 Düsseldorf, Germany; Urological Ambulatory PandaMED, Alleestrasse 105-107, 42853 Remscheid, Germany

**Keywords:** Haemorrhagic cystitis, Radiotherapy, Cyclophosphamide, Hyperbaric oxygenation

## Abstract

**Background:**

Radiotherapy and cyclophosphamide-induced haemorrhagic cystitis are rare but severe complications occurring in 3–6% of patients. Hyperbaric oxygen treatment (HBOT) has been demonstrated to be an effective treatment for haematuria not responding to conventional management. Only very few data exist for long-term follow-up after HBOT.

**Methods:**

We retrospectively reviewed 15 patients referred for HBOT for haemorrhagic cystitis (HC). HBOT was performed for 130 min/day at a pressure of 2.4 atmospheres. We evaluated patient demographics, type of radio- and chemotherapy and characteristics of haematuria. The effect of HBOT was defined as complete or partial resolution of hematuria according to the RTOG/EORTC grade and Gray score.

**Results:**

A total of 15 patients (12 after radiotherapy, two after chemotherapy and one patient with a combination of both) were treated with a median of 34 HBO treatments. Radiotherapy patients received primary, adjuvant, salvage and HDR radiotherapy (60 – 78 Gy) for prostate, colon or cervical cancer. The patient with combination therapy and both of the chemotherapy patients were treated with cyclophosphamide. First episodes of haematuria occurred at a median of 48 months after completion of initial therapy. The first HBOT was performed at a median of 11 months after the first episode of hematuria. After a median of a 68-month follow-up after HBOT, 80% experienced a complete resolution and two patients suffered a singular new minor haematuria (p < 0.00001). A salvage-cystectomy was necessary in one patient. No adverse effects were documented.

**Conclusions:**

Our experience indicate that HBOT is a safe and effective therapeutic option for treatment-resistant radiogenic and chemotherapy-induced haemorrhagic cystitis. For a better evaluation prospective clinical trials are required.

## Background

Radiotherapy-induced haemorrhagic cystitis (RHC) after pelvic radiation therapy is a rare but known long-term complication which develops in 3–6.5% of patients. RHC can occur from 6 months to 20 years after radiation therapy [1-3]. Chemotherapy-induced haemorrhagic cystitis (CHC) can also cause chemotherapy-limiting haematuria (cyclophosphamide or others oxazaphosphorines) [4-7]. Since the introduction of Mesna (2-mercaptoethanesulfonic acid), a thiol compound, for uroprotection the incidence of CHC has decreased to less than 5% [5]. Despite the underlying cause, HC is a relevant impairment of the patients’ quality of life with often multiple and mostly emergency admissions to hospitals.

As for other severe haematurias, bladder irrigation is the primary treatment for HC. Different systemic or intravesical agents such as hyaluronic acid, aminocaproic acid, formalin or prostaglandins have been used, however with limited success [8]. As a consequence, bladder fulguration is repeatedly used as a therapeutic option to stop bleeding and blood is transfused when indicated. In the most severe cases selective embolization of the hypogastric arteries or salvage cystectomy with urinary diversion may be necessary [9].

Hyperbaric oxygen therapy (HBOT) is a promising but still extreme rarely-used therapeutic option for patients in whom the standard management has failed. By increasing the tissue oxygen level by up to 15-fold, HBOT promotes capillary angiogenesis which also increases the regeneration of damaged urothelium. Studies report success rates of 73 to 96% [1,3,10-13]. We present here our results of HBOT for the treatment of RHC and CHC.

## Methods

We retrospectively reviewed all 23 patients treated with HBOT for haemorrhagic cystitis between 01/2002 and 10/2014 in two urological departments. In 15 patients a complete follow-up was achievable. Patient demographics were recorded, as was for RHC the total radiation dosage and the type of radiation therapy. Follow-up was ensured by outpatient contacts in the clinic and by cooperating ambulatories. Each patient gave informed consent and the study was approved by the institutional review committee of Witten/Herdecke University.

### Evaluation of hematuria

Initially, all patients underwent permanent bladder irrigation and evacuation of blood clots. Urine cultures were set up before irrigation to detect infectious haematuria. When urine cleared after bladder irrigation patients underwent a cystoscopy or, in cases of heavy bleeding, a transurethral resection / fulguration to rule out an urothelial carcinoma (including carcinoma in situ). Additionally, the upper urinary tract was evaluated to exclude it as a source of bleeding.

The onset of the first relevant haematuria and the maximum severity of all haematuria episodes were evaluated. The grading of haematuria was determined from the RTOG/EORTC grade for RHC, and the Gray score for CHC [14,15] (Table 1). The difference in haematuria grade before and after treatment was analyzed by using a paired t-test. Statistical significance was selected as a value of p < 0.05.

**Table 1:** **Classification scores for radiotherapy and chemotherapy induced haemorrhagic cystitis: RTOG/ EORTC and Gray scores (modified from** [14] **and** [15]**)**

**Classification scores for chronic radio- and chemotherapy induced haemorrhagic cystitis**					
**Radiotherapy**	**RTOG/ EORTC scale**	**Grade 1**	**Grade 2**	**Grade 3**	**Grade 4**
		Microscopic haematuria, requiring no medication; Minor telangiectasia	Moderate haematuria requiring medication; Moderate telangiectasia	Severe haematuria; Severe telangiectasia	Necrosis; Severe haemorrhagic cystitis
**Chemotherapy**	**Gray score**	**Grade 0**	**Grade 1**	**Grade 2**	**Grade 3**
		Normal, no haematuria	Mild haematuria with telangiectasia or dilatation of the bladder vessels	Severe haematuria with mucosal haematomas	Severe haematuria with intravesical clots

### Hyperbaric oxygen therapy

Hyperbaric oxygen therapy was initiated when conventional therapy options remained unsuccessful and when no contraindication for HBOT was given (assessment with otoscopy, spirometry and ECG). We evaluated the time between the first haemorrhagic episode and the beginning of HBOT, the number of HBOTs and all HBOT-related data (Table 2).

**Table 2:** **Clinical characteristics of patients and details of CHC and RHC and HBO treatment**

**Sex**	**Age/Therapy**	**Age/End of Follow-up**	**Diagnosis**	**Treatment**	**HBO IndicationIndikation**	**End of Therapy**	**Time till Haematuria [m]**	**RTOG/Gray before HBOT**	**Admissions to Hospital**	**Number blood transfusions**	**Duration till HBO [m]**	**HBO Treatments**	**Follow Up [m]**	**Events/Follow-up**	**RTOG/Gray after HBO**
M	68	70	PCA	adj	RC	08/2008	34	3	5	0	4	40	33	no relapse	0
M	82	86	PCA	prim	RC	07/2004	54	3	3	0	15	24	53	no relapse	0
M	73	81	PCA	HDR	RC	08/2002	11	4	4	2	33	24	100	1x haematuria 2007	3
W	71	83	CCA	adj	RC	07/1997	30	3	3	0	28	128	142	no relapse	0
M	80	86	PCA	prim	RC	07/1998	5	4	2	2	110	40	78	no relapse	0
M	71	76	PCA	adj	RC	01/1999	76	4	2	1	36	35	28	no relapse, †2010	0
W	86	90	UCA	adj	RC	01/1990	234	4	3	2	0	6	29	no relapse, †2012	0
M	80	83	PCA	adj	RC	01/2000	124	4	3	3	1	51	16	SRC 2010, †2012	4
M	65	72	PCA	adj	RC	03/2000	50	4	3	1	30	26	94	no relapse	0
M	61	63	PCA	sal	RC	02/2010	20	4	3	4	7	20	133	no relapse	0
M	22	33	RPSS	comb	RC/CC	01/1999	43	4	2	12	11	34	28	no relapse	0
M	73	78	PCA	sal	RC/RP	07/2007	14	3	2	2	1	48	68	no relapse	0
M	65	71	PCA	adj	RC/RP	07/2003	48	4	3	2	3	20	49	no relapse, †2011	0
W	49	56	LE	prim	CC	01/1974	362	3	4	0	40	40	86	1x haematuria 2008	3
M	39	49	WG	prim	CC	01/1993	120	4	3	1	4	17	136	no relapse	0

†=deceased.

**Figure 1:**
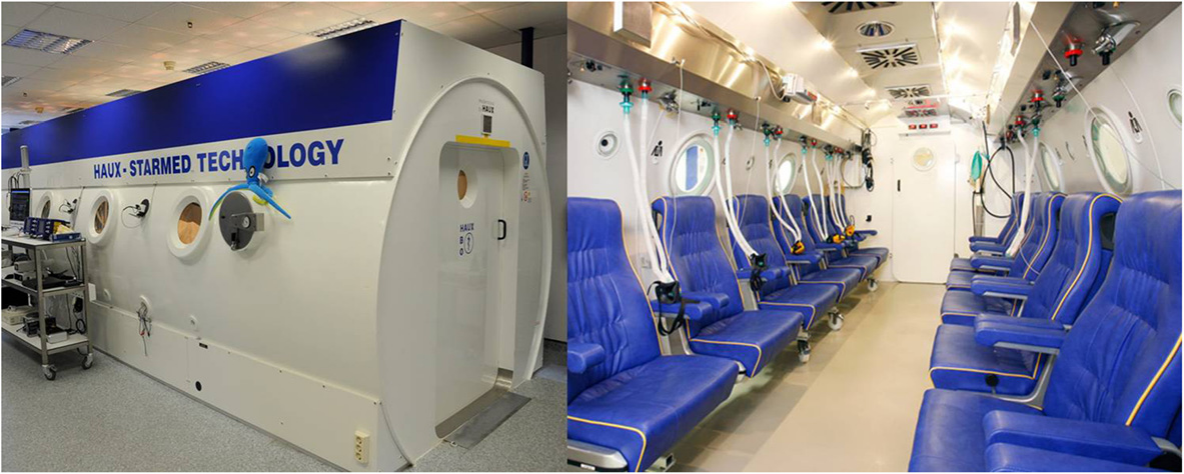
Exterior and interior view on the pressure chamber for max. 12 persons.

**Figure 2:**
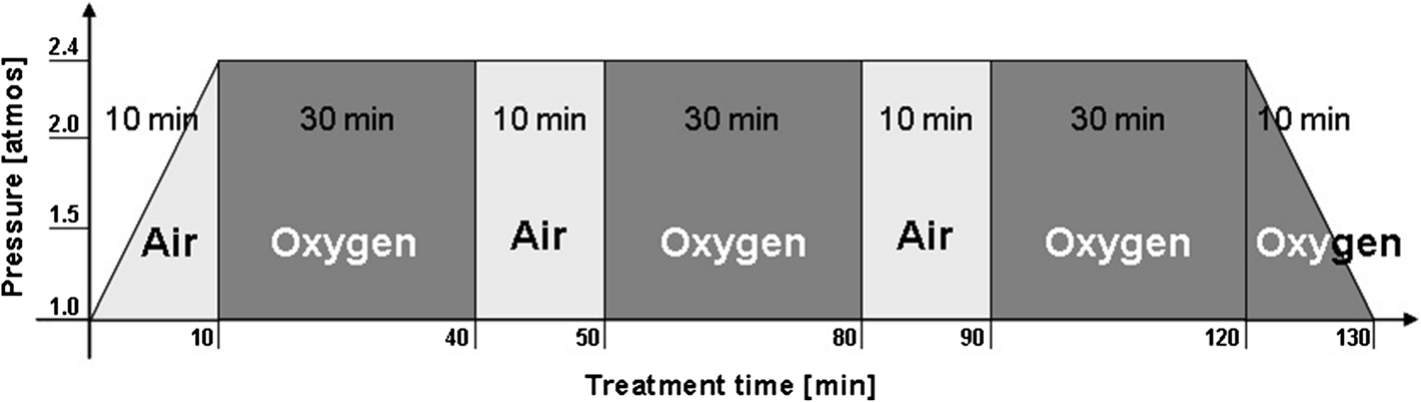
Diagram of the therapy scheme TS 240–90. “Air” is defined as normal ambient air with 21% (v/v) oxygen. “Oxygen” is defined as the application of 100% (v/v) oxygen. The pressure [atmosphere] is plotted on the y-axis, the treatment time [min] on the x-axis (adopted from [24]).

All patients were treated in the same HBOT centre where HBOT was performed in a treatment chamber with a capacity of up to 12 patients (Haux Starmed, HAUX-LIFE-SUPPORT GmbH, Karlsbad-Ittersbach, Germany) (Figure 1) under continued monitoring. The therapy scheme TS240-90 [16] is composed of 3x30 minute periods of pure oxygen (at a pressure of 2.4 atmospheres). The episodes of pure oxygen were interrupted by 10 minutes with normal air (21% (v/v) oxygen) to reduce oxygen toxicity (Figure 2). The increase of the partial pressure of oxygen (pO_2_) was measured percutaneously. HBOT was performed five days a week. After each HBOT session the grade of haematuria was evaluated and patients were checked for barotraumas.

The effect of HBOT was defined as complete (no further macro-haematuria until completion of follow-up) or partial resolution of haematuria (defined as a lower RTOG/EORTC or Gray score than before HBOT).

## Results

In 15 patients (12 males, 3 females) undergoing HBOT for therapy-resistant haemorrhagic cystitis between 2002 and 2014 a complete retrospective follow-up was achieved. Additionally, two patients suffered from haemorrhagic proctitis. At the beginning of HBOT the age ranged from 22 to 86 years (mean age 71). At the end of follow-up the age ranged from 33 to 90 years (mean age 76 y).

Radiotherapy was performed owing to prostate (10 patients, 67%), colon or cervical cancer (1 patient each, 7%). One patient underwent combined radio-chemotherapy owing to retroperitoneal synovial sarcoma (with Ifosfamide, ISF) and two patients (13%) were treated with cyclophosphamide for Wegener’s granulomatosis or Lupus erythematosus.

In 7 patients (47%) radiotherapy was performed as an adjuvant because of prostate, colon or cervical cancer (60–64 Gy). Primary and salvage radiation was applied to two patients (13%) for prostate cancer (66 and 70 Gy) and one patient (7%) was treated with HDR brachytherapy (78 Gy) for prostate cancer. The radiation dose of the patient with retroperitoneal synovial sarcoma was 66 Gy.

The first episodes of haematuria occurred between 5 and 362 months (median 48 months) after the beginning of radiation or chemotherapy. A median of three (2 – 5) admissions to the hospital and up to seven outpatient treatments were necessary. According to the RTOG/EORTC score haemorrhagic complication Grade 4 occurred in nine patients (60%) and Grade 3 in four (27%). Patients with CHC had haemorrhagic complication Grade 3 or 4 respectively (Gray score). Blood transfusions (between 1 and 12 units) were necessary in 11 patients (73%).

In all patients urothelial malignancy was excluded by histology. At least one bladder fulguration was performed in every case in order to stop the bleeding but no patient received any intravesical instillation therapy.

When HC was resistant to conventional therapy (without instillation therapy) HBOT was initiated, in a median of eleven months (0 – 110) after the first episode of haematuria. Between 6 and 128 HBOT sessions (mean 34) were necessary to achieve a total resolution of haematuria. In all HBOT sessions the partial pressure of oxygen measured percutaneously was increased to 1250–1480 mmHg.

Follow-up ranged from 16 to 142 months (mean 68). In 12 patients (80%) a total resolution of the HC could be achieved without any further haematuria. In two patients (13%) bladder irrigation had to be re-performed once for a singular recurrence of haematuria (RTOG/EORTC Grade 3). Changes in RTOG/EORTC scores were statistically significant (p < 0.00001).

In one case a salvage cystectomy with urinary diversion (ileum conduit) was necessary owing to a fulminant bleeding episode. The overall success rate was 93%. The radiogenic proctitis completely recovered in both cases. No side effects were noted in any patient.

## Discussion

Radiation and cyclophosphamide-induced HC is a severe and potentially life threatening complication. Management can be highly challenging with well-known urological treatment options. It is to be expected that the number of radiation therapies will increase during the next decades [17]. In parallel a rise in complications and side effects, such as RHC, can be expected. The results of this study suggest that HBOT is a promising option in therapy-resistant HC with a success rate of more than 90%.

In both CHC and RHC the acute reaction on urothelial damage is an inflammatory response causing oedema, ulceration, neovascularisation and haemorrhagic necrosis. This is usually limited to the treatment period.

In CHC urotoxicity is caused directly by the renal excretion of acrolein, metabolites of oxazaphosphorine alkylated drugs. There is no clear dose-related relationship. Since the 1970s the prophylactic application of Mesna has been used as a standard protocol for prevention of haemorrhagic complications. It neutralises acrolein and its toxic effects to the urothelium. The incidence of CHC is reduced from about 68% to about 5% [5].

In RHC urothelial damage is caused by radiolysis of water. The concentration of activated free oxygen radicals increases; as these are highly reactive, they cause cell membrane injury by lipid peroxidation, which induces immediate cell death. Additionally, direct (by radiation energy) and indirect (by oxygen radicals) DNA damage causes replication failures resulting in delayed cell death [8]. This damage is dose-related.

This acute reaction can escalate into a chronic endarteritis which seems to be the determining pathology of late-onset RHC and has been described as the “three-H model”: the progressive endarteritis and vascular rarefaction (**h**ypovascular) results in critical ischaemia (**h**ypoxia) with a reduction in oxygen concentration of up to 70-80%. Ischaemia of the mucosal tissue can lead to necrosis and sloughed off cells because of impaired healing capacity. A compensatory teleangiectasia develops and causes (persistent) haematuria. Finally, fibrotic repair processes can lead to impaired bladder capacity (**h**ypocellular) [3,11]. This process can still become relevant up to 20 years after radiation therapy [2]. In our study haematuria occurred in a median of 41 months after completion of radiation therapy. This is within the range of previously described periods of 35–48 months [1,2,8,10].

Tissue oxygen supply occurs primarily via diffusion from the capillaries by significantly increasing the physically dissolved oxygen in the plasma (100% O_2_ pressure of 1.4 atmosphere). HBOT improves the local and regional tissue oxygen supply. Our results demonstrate a drastically increased pO_2_ in the tissue from 1250 to 1480 mmHg from percutaneous measurements. By increasing pO_2_, macrophages, fibroblasts and granulocytes may resume their normal function and mediate repair processes. In addition, HBOT directly induces neo-angiogenesis, whereby 80% of the normal capillary density can be achieved. Furthermore HBOT causes anti-oedematous vasoconstriction without secondary ischaemic hypoxia [18-20].

Hyperbaric oxygen in patients with severe RHC or CHC not responding to conventional urological treatment is an extremely rare and not yet standardised treatment option.

In RHC different research groups and we have reported success rates between 75 and 96% in both prospective and retrospective study designs [10,13,19,21-23]. In contrast to our study, other cohorts have varied between 13–60 patients with only short follow-up periods of 12–30 months.

We could demonstrate a success rate of 90% even after a median long-term follow-up period of 68 months, confirming the excellent long-term results reported by Nakada et al. whose success rate was 75-88% [12].

Only a few cases patients with CHC have been reported. One of the larger series with six CHC patients reported a 100% success rate after 11 to 36 months of follow-up (although they noted that in one patient haematuria recurred after three months) [24]. In other reported case reports a complete loss of haematuria occurred after a follow-up period of 11–36 months [7,24-26].

The main advantage of this therapy is that there are no severe side-effects. The most common adverse effects of HBOT are middle ear or sinus barotraumas requiring myringotomy in up to 5% [27]. Reversible myopia has also been described as a dose-dependent HBOT side effect [27]. Our study, as many other reports, did not show any adverse effects of HBTO.

So far variable success of HBOT has been discussed without any clear results, mainly because of the small sample size in most series.

In RHC the total radiation dose seems to have an influence on HBOT success: generally, complications such as RHC begin at a total dosage of 45–55 Gy and increase significantly at a cumulative dose of ≥60 Gy (≥5% with late effects) [28]. Nakada et al. showed a significantly better HBOT success in patients exposed to lower radiation doses (62Gy vs. 76Gy, p < 0.001). In addition del Pizzo et al. reported relatively poor HBOT long-term success rates (27% after 5 years) in patients with high dosage radiotherapy (75Gy) [29]. In the present study most of the patients received a cumulative dose between 60 – 66Gy. One patient with high dose therapy (78 Gy) suffered one unique relapse of haematuria.

Some authors discuss that there might be an influence of the number of HBOT sessions on the therapeutic success in RHC. Most of the studies applied between 30 and 40 treatment sessions [13,19,21,29] which is comparable to our data with a median of 34 treatments. The significantly higher number of HBOTs (62) may be the reason for the excellent long-term success in Nakada’s study compared with a study with a success rate of 64% after only 14 HBOTs [30]. The few existing data suggest that fewer HBOTs are sufficient in CHC patients. The reported series of six CHC cases required a median of 27 HBOTs, other published cases between 14 and 40 treatments [7,24-26].

The period of time between the onset of haematuria and the beginning of HBOT is discussed as another success factor for RHC. In some studies, patients with a shorter pre-treatment interval (6–8 months) showed significantly better results on HBOT (p <0.001) [12,13]. We could not demonstrate any relationship between the time to beginning of HBOT and its success. This is most probably because, as CHC is the more acute event, HBOT was started at a mean of 47 days after the onset of symptoms. However, intervals of up to four months between first admission and the beginning of HBOT have been described [24].

Finally the influence of patient age is controversially discussed. Some studies suggest that younger patients (≤70 years) have higher success rates. Other studies, as well as ours, have not found any correlation between age and HBOT success. Furthermore, in CHC patients a complete remission of haematuria is reported in cases aged from 15 to 82 years [1,12,13,24-26].

The costs amount to approximately €200 per HBOT session. Accordingly, the median costs per patient in this study were €6,800 (34 HBOT sessions). The costs are comparable to a cost-analysis case study from Australia which showed HBOT to provide major health cost savings [31].

Our study, as many others, is certainly limited by the small study population and the retrospective design. Furthermore different treatment protocols reduce the comparability of treatment outcomes. Prospective trials would be desirable for further evaluation but the first planned prospective and randomized study (initiated by the Baromedical Research Foundation) was cancelled because of poor recruitment (NCT00134628). Nevertheless, we believe that our results show the long-term efficiency and safety of this therapy option in patients suffering from radiation- and cyclophosphamide-induced haemorrhagic cystitis.

## Conclusions

Our data suggest that hyperbaric oxygen therapy is a safe and simple therapy option with a long-term success rate of approximately 90% with hardly any side-effects. Therefore HBOT should be considered more often in cases of severe haemorrhagic cystitis induced by radiation and/or cyclophosphamide.

## Abbreviations

HC: Hemorrhagic Cystitis
HBOT: Hyperbaric Oxygen Treatment
RHC: Radiotherapy-induced Hemorrhagic Cystitis
CHC: Chemotherapy-induced Hemorrhagic Cystitis
RTOG: Radio Therapy Oncology Group
EORTC: European Organisation for Research and Treatment of Cancer
ECG: Electrocardiography
PCA: Prostate Cancer
CCA: Colon Cancer
UCA: Uterine / Cervical Cancer
RPSS: Retroperitoneal Synovial Sarcoma
LE: Lupus Erythematosus
WG: Wegener’s Granulomatosis
Adj: Adjuvant Radiation
Prim: Primary Radiotherapy
HDR: High-Dose Rate
Sal: Salvage Radiotherapy
Comb: Combined Chemoradiation
RC: Radiotherapy-induced Cystitis
RP: Radiotherapy-induced Proctitis
CC: Chemotherapy – induced Cystitis
SRC: Salvage Cystectomy
